# An Assessment of Social Media Usage Patterns and Social Capital: Empirical Evidence From the Agricultural Systems of China

**DOI:** 10.3389/fpsyg.2021.767357

**Published:** 2021-10-28

**Authors:** Gui-hua Xie, Lin-ping Wang, Asif Khan

**Affiliations:** ^1^College of Economics and Management, Fujian Agriculture and Forestry University, Fuzhou, China; ^2^College of Business Administration, Fujian Business University, Fuzhou, China; ^3^Department of Marketing and Distribution Management, College of Management, National Kaohsiung University of Science and Technology, Kaohsiung, Taiwan

**Keywords:** social capital, social media usage, agricultural systems, agricultural entrepreneurs, entrepreneurship training, digital transformation, sustainable education and learning, sustainable agriculture

## Abstract

This research offers a theoretical model to measure the impact of social media usage on social capital in the agricultural system of China. Furthermore, this research also investigates the relationship between agricultural policies related to entrepreneurship training and social media usage. A total of 589 questionnaires were distributed in the training courses of Fujian Agricultural Vocational Technology College, and, Fuzhou, Quanzhou, Jianning, and Liancheng counties and cities in Fujian during winter and summer vacations to target new vocational farmers. The results show that social use, hedonic use, and cognitive use of social media significantly impacted both bridging and bonding social capital. Furthermore, the results of the study suggest that entrepreneurs who have participated in the training have significantly higher levels of social use and cognitive use than those who have not been trained. The findings of this paper have implications for the digital transformation by agricultural entrepreneurs in recognition of the role of sustainable education and learning in entrepreneurial activities and the utilization of social and cognitive functions of social media to acquire and accumulate social capital and provide support for sustainable agriculture and rural development. Furthermore, the concepts of sustainability-driven agriculture in the digital transformational framework were also studied and it was indicated that transformed agriculture can effectively deal with the present challenges.

## Introduction

To successfully overcome the issues and attain the objectives related to the ideas of sustainable advancement, businesses and disruptors across the globe should recognize the crucial drivers of digital transformation that can influence businesses and industries (Lammers et al., 2018). Digital transformation empowers the continuance of the growth of the economic and human social development of countries. This enhances the quality of life of people that is attained by the impartial allocation of all benefits, safeguard from unavoidable adverse impacts, and guaranteeing the development of the abilities of all the country’s residents. Agriculture, innovation, services, and industry, powered by digital technologies, are revealed as four vital elements of economic growth worldwide. At present, agriculture is viewed as an important component of the value chains, offering valuable services to humanity, safeguarding food safety measures, strengthening and improving countries’ financial systems, and its expansion concentrates on social, environmental, and economic sustainability (Hrustek, 2020). The digital transformation in the agricultural industry has several advantages to the agricultural entrepreneurs, involving enhanced market clarity, greater farm efficiency, and more economical logistics. In specific, under the public health issues produced by the COVID-19 pandemic, digital agriculture has aided emerging economies to survive the pandemic’s unfavorable impacts on supply chains and food production (Xie et al., 2021).

Corporations are progressively utilizing social media (SM) as a proper channel for communication that is revolutionizing the methods corporations use to manage suppliers and customers (Pekkala and van Zoonen, 2021). Information, change, and diversity are considered to be the most significant factors in the modern world; consequently, this period is known as “the era of revolution in knowledge and interaction.” The variety of information and the velocity of flow of data are amongst the factors that portray this period as unique. Hence, this period can be described as “the era of information.” Despite having many descriptions, the main purpose of this era is to boost social networking and develop an information and knowledge-based society. Considering the diversity of current technological advancements, building a competitive edge needs innovative reforms at each phase of life. For instance competition in institutions is based not only on knowledge, skill, and capability but also on ideas like resolution, business, unity, respect, and progress. Contemplating these recent fundamental alterations in the corporate world, social media has a significant position as changing internet utilization conduct and marketing strategies (Habibi et al., 2014). Numerous companies around the globe including universities, airline businesses, banks, and GSM operators boost their marketing and advertising strategies with the help of social media (Genç and Öksüz, 2015). These findings suggest that there are relatively large differences in the adoption and utilization of social media among different groups and that the effects on social capital vary. Research on social media used by specific groups is useful for exploring the impact of social media on human activities. Agricultural entrepreneurs are related to the development of modern agriculture, and they are considered to be one of the specific groups utilizing social media, and hence they have received increasing attention from research scholars. A study by Murray (2015) found that social media is beneficial in increasing farmers’ social capital and thus, it can lead to enhance transparency, engagement, and authenticity in the supply chain. This study signifies three crucial aspects of SM usage including, social use, cognitive use, and hedonic use. These factors relate to, correspondingly, the usage of social media to develop and sustain social relationships; the usage of SM for pleasure and relaxation; and the usage of social media to produce and deliver user-created content (Ali-Hassan et al., 2015).

Social capital (SC) signifies reserves or resources embedded in a person’s or company’s system of social relationships. This study implements Adler and Kwon’s (2002) description of SC that is centered on a broad exploration of the literature. Corresponding to this description, social capital can be defined as “the goodwill offered to people or companies. It is based on the formation and sustainability of the individuals’ social relationships. It has an impact on the flow generated from the knowledge, impact, and unity it provides to the individuals.” A reassessment of a relatively current social capital research by Kwon and Adler (2014) suggests that the idea has grown and developed into an entire area of research. The study further claims that most findings from diverse areas such as organizational and management study, the fundamental theory for this description is no more in disagreement. A conventional method for determining social capital concentrates on organizational social capital and explores the evolving bonding and bridging relations (Putnam, 2000). Ellison et al. (2007) built a framework to explore the correlation between the use of Facebook and bonding and bridging SC. Furthermore, Koroleva et al. (2011) also targeted social media research and provided a further comprehensive viewpoint of social capital by concentrating on the distinctive advantages it holds. Social capital in the framework of rural and agricultural innovation is commonly stated to allow accessibility to supplies including funding, information, and moral assistance that can help to promote agricultural innovation (Tregear and Cooper, 2016). According to Micheels and Nolan (2016), social capital is comparatively more essential than the size of the farm in deciding the types of strategies and technologies to be implemented by the firm. Moreover, the three distinct types of SC including bridging, linking, and bonding social capitals offer solutions for various kinds of relationships (Cofré-Bravo et al., 2019). Social capital theory differentiates the characteristics related to the type of relations between bridging, linking, and bonding social capital. Social capital has been frequently implemented to identify innovation systems (Micheels and Nolan, 2016; Saint Ville et al., 2016; Hunecke et al., 2017) studies in agricultural settings and information exchange (Michelini, 2013; Tregear and Cooper, 2016). This study aims to cover the research gap, by examining the relationship of three types of social media usage, namely, social, hedonic, and cognitive orientation usage with bonding and bridging social capital of agricultural entrepreneurs.

As a socioeconomic component, entrepreneurship is considered to be the most essential element related to the economic revolution of the world, and its impact on several regions of socio-economic relationship is undeniably massive. Entrepreneurs’ technical learning and training is a constant course. Hence, it practically signifies the idea of entrepreneurship as an action built on the formation of circumstances, suitable for attaining the determinations that are intended at improving the standard of living of the people and obtaining appropriate economic and ethical impact. Periodicity is a characteristic of entrepreneurship, and it involves the constant exploration for improvements and innovations. Entrepreneurship goes through the phases of the growth life cycle and certainly converts into larger or new business ideas and innovative entrepreneurial strategies. That is precisely the explanation regarding the consideration of exploring and investigating skills as the fundamental professional expertise of an entrepreneur (Shindina et al., 2015). The current business environment demonstrates a variety of aspects related to small and medium businesses including the relationships between knowledge and networks. The achievement of small enterprises depends on developing “dedicated” relations; and hence, they can utilize the opportunity provided by social media in building significant relationships (Schaffer, 2013). Consequently, social media, due to its dynamic, equivalent, consistent, and collaborative nature presents prospects to all entrepreneurs and organizations to train themselves and take benefit of these widespread interaction tools for their businesses (Genç and Öksüz, 2015). This research aims to examine the significant differences between social media social use patterns of the agricultural entrepreneurs with and without entrepreneurial training experiences.

This study offers theoretical and practical contributions. The findings of this research represent a novel theoretical contribution connecting social media (SM), social capital (SC), and entrepreneurial training. This research study provides a theoretical model to measure the impact of three dimensions of SM usage including social, hedonic, and cognitive use adapted from use and gratification (U&G) theory with two dimensions of SC consisting of bonding and bridging social capital adapted from social capital theory. Furthermore, this research also investigates the correlation between entrepreneurial training and social media usage. Additionally, it will help to understand people’s social media usage patterns and provide guidance and reference for the formulation of government entrepreneurship training policies and the effective use of SM tools for agricultural entrepreneurs. This research covers at least two major research gaps. First, it examines the relationship of the three dimensions of social media usage on social capital. Second, it explores the impact of entrepreneurial training on social media usage.

## Theoretical Background

### Social Media Usage Patterns

Use and gratification theory (use and gratification) believes that people will choose the media they want to use according to their needs and use different functions of the media in different contexts (Ruggiero, 2000). Technology utilization is at the core of several frameworks describing the recognition, influence, and significance of information systems. However, a crucial drawback of these technological frameworks is based on their homogeneous strategies and conduct for the utilization of technology including frameworks frequently being criticized for handling usage activities generally (Barki et al., 2007; Venkatesh and Bala, 2008). This critique becomes further significant as technology progresses and contributes itself to various usage activities and environments, as in the case of SM. This study utilizes the uses and gratification (U&G) theory to investigate the environment of social media usage within businesses. Uses and gratification study includes the societal and emotional roots of demands, which produce prospects of the media and control the various forms of media coverage or involvement in other actions, causing the gratification of needs (Katz and Kahn, 1978). A principal hypothesis of the uses and gratification theory is that media usage is driven by a person’s logical needs self-consciousness and belief related to the satisfaction of the needs via specific forms of content and media. Conventional uses and gratification research have recognized three main kinds of needs, which can be satisfied by the use of a diverse set of media. For instance, social needs are defined as the desire to improve interaction with friends, acquaintances, and family. Whereas hedonic needs are described as emotional needs for enjoyable and pleasurable encounters; and cognitive needs are illustrated as the need to obtain knowledge, expertise, and insight (Ali-Hassan et al., 2015). Research studies have discovered evidence for all three types of needs via SM usage (Quan-Haase and Young, 2010; Papacharissi and Mendelson, 2011; Whiting and Williams, 2013; Ali-Hassan et al., 2015).

In this article, we theorize three elements of SM usage that relate to the needs mentioned earlier: social use, hedonic use, and cognitive use. Social use refers to agricultural entrepreneurs’ usage of social media to make friends, create new social relationships, and staying in contact with current friends and acquaintances for social interaction (Papacharissi and Mendelson, 2011). Hedonic use refers to agricultural entrepreneurs’ usage of social media for recreational purposes like playing games, watching videos, listening to music, and other activities. Cognitive use refers to the production and distribution of agricultural content on social media and retrieving content generated by other people to enhance their own and others’ perceptions, including sharing text, images, and video messages, posting their own opinions, stories, comments, and likes, and viewing other people’s content on social media (Ali-Hassan et al., 2015).

### Social Capital

The social capital theory is currently broadly utilized in exploring agricultural and rural innovation (Fisher, 2013; Tregear and Cooper, 2016; King et al., 2019). Previous studies in SC incorporate important research, like the study of James Coleman. According to Coleman (1988), as physical capital is formed by adjustments in resources to create tools that accelerate manufacturing, human capital is formed by alterations in individuals regarding their abilities and skills that enable them to perform in innovative approaches. Social capital, nevertheless, is generated by alterations in the relationships between people that enable engagement. Physical capital is tangible and can be represented in visible material shape, however, human capital is not as tangible and can be represented in the abilities and expertise developed by a person, whereas social capital is comparatively less tangible because it occurs in relationships. Woolcock and Narayan (2000) underline the significance of social capital for businesses. They state that the fundamental notion of social capital is that an individual’s friends, family, and acquaintances represent an essential resource, which can be relied upon in the time of crisis, appreciated for its likeliness, and leveraged for significant gain. The concepts related to individuals are also applied in the similar way to groups as well. Likewise, Putnam (2000) discovers that SC is the combination of values, networks, and social trust that individuals can utilize to enhance their standard of living and to carry out common goals. The mentioned studies shared a commonality of linking social capital to social arrangements, and trust in networks that are conveyed differently in various kinds of social capital (Cofré-Bravo et al., 2019).

Social capital encompasses the optimistic advantages that are obtained from social interactions. Social capital performs a crucial part in deciding the flows of information, and it additionally involves managerial knowledge. Companies have a lead over the competition in the design and transmission of information since companies are extra efficient in creating high degrees of social capital. SC can be split into bridging and bonding capital. Bonding capital is rooted in the associations of deeply attached or standardized groups that regularly trade strong emotive and fundamental assistance. Bridging capital rests in the interactions of weakly tied people with heterogeneous backgrounds. Such capital helps people broaden their societal possibilities and develop their prospects to obtain innovative resources and knowledge. The weak-tie theory claims that pervasive weaker relations that derive from bridging capital are usually extra valuable for developing innovative concepts as compared to the strong interactions of bonding social capital (Shang and Sun, 2021). Individuals bridging the underlying gaps—that is, an absence of an immediate interaction or connection among two or more individuals—can retrieve more knowledge flows and attain stronger advantages from the network (Burt, 1997).

In the mobile Internet context, studies on general Internet users and corporate employees have found that social media has a substantial influence on the growth of social capital, either positively or negatively, due to its network and communication characteristics. In the agricultural field, there is a shortage of investigation on the impact of SM on overall social capital, except for a few case studies and studies on social trust. In this research, the classification suggested by Putnam (2000) is utilized to categorize the social capital of agricultural entrepreneurs into bridging SC and bonding SC and focus on the relationship between specific media The relationship between specific media usage and these two kinds of social capital can help enrich the study on the association between and social capital. The bridging SC discusses the possibility of communication, the occurrence of communication, level of confidence, and exchange shaped by the network of interaction among agricultural entrepreneurs and their friends, and relatives in the agricultural entrepreneurial team. On the other hand in this study, the bridging SC is described as, the social capital shaped by the network of diverse relationships among agricultural entrepreneurs and customers, suppliers, government, society, media, and agents in the peripheral environment.

## Hypothesis Development

### Social Orientation Usage and Social Capital

Previous research has shown that the usage of SM to create and sustain social relationships directly or indirectly can significantly influence bonding social capital (Wu, 2013). The Internet is a constructive force that serves as an instrumental bond that can help individuals develop expressiveness (Anderson and Rainie, 2010). Correspondingly, according to the findings of Ellison et al. (2007), a connection was discovered between the usage of SM and the growth of communicative relationships. Even without meeting or contact, social media can maintain people’s understanding of each other as well as traditional media such as the telephone. Cao et al. (2012) discovered that utilizing social networking sites at a job to retain and enhance interaction with associates had a significant impact on trust between colleagues. Furthermore, Ellison and Vitak (2015) concluded that the usage of corporate social networking sites uncovered coworkers’ social networks, which in turn leads to “identity assurance” and provides signals of credibility and trust-building. For agricultural entrepreneurs, the emergence of social media has facilitated communication and contact between friends, family, and employees within the team on the issues specific to agricultural operations, enhancing bonding, trust, and team cohesion. Synthesizing the above literature, the following research hypotheses were derived.

H1a: Social orientation usage of social media has a significant positive effect on the bonding social capital of agricultural entrepreneurs.

Ellison et al. (2007) argue that technology can enable casual connections between people and build bridges in the workplace. Social media is influential as a mobile communication tool, and in addition to the above technological roles, it also has a well-developed search engine and managerial features that let users discover other people and create relationships. Liu et al. (2016) argue that the usage of SM is effective and powerful for the acquisition of bridging social capital because social networks allow people to interact with a comparatively large number of people, even if most of them have never met each other in person. Numerous studies have shown that social networking services are good for building weak ties, which are the basis for constructing social capital. In agricultural entrepreneurship, social media communities, the information shared can help agricultural entrepreneurs quickly find people sharing common interests or meet their entrepreneurial needs to expand their connections. At the same time, SM allows people to build and maintain weak relationships inexpensively. Because of the low cost of social media use, agricultural entrepreneurs can maintain a large and dispersed network of relationships at a low cost and leverage them to attract resources. Taken together, the above literature leads to the following research hypotheses.

H1b: Social orientation usage of social media has a significant positive effect on bridging the social capital of agricultural entrepreneurs.

### Hedonic Orientation Usage and Social Capital

Scholars have discovered that the hedonic use of SM, unlike previous inventions of technology, does not lead to societal segregation and supports the creation of “electronic relationships” (Colwell and Payne, 2000). Social media-based entertainment is characterized by interactions with other people that can positively affect users’ fundamental social capital by growing the number of social relationships, as people are inclined to play with friends or become friends with the people they are playing games with (Longman et al., 2009). In the case of agricultural entrepreneurs, rural communities are poor in recreational facilities, agricultural entrepreneurial activities are full of hardships, and daily labor does not allow them many opportunities to engage in recreational activities. However, they can reap the benefits of SM because social media is easy to use, and furthermore, they can use it to watch videos, learn about entertainment news, listen to music, or play popular games with friends, be up to date with the trendy fashion topics, and maintain connections with friends. Integrating the above literature, the following research hypotheses can be derived.

H2a: Hedonic orientation usage of social media has a significant positive effect on the bonded social capital of agricultural entrepreneurs.

Similarly, social media usage for hedonic purposes can also offer prospects for “opportunity relationships” and the creation of new connections between individuals (Ellison et al., 2007). According to the findings of Cole and Griffiths’ (2007) study related to hedonic use and social capital, it was found that online gaming environments provide opportunities to build emotional relationships and form friendships. Agricultural entrepreneurs often use the entertainment function of social media to find friends with common interests and continuously expand their circle of friends. Hence, this research investigates the impact of the hedonic use of social media on the bonding and bridging social capital of agricultural entrepreneurs. Consequently, the following research hypotheses can be postulated.

H2b: Hedonic orientation usage of social media has a significant positive effect on the bridging social capital of agricultural entrepreneurs.

### Cognitive Orientation Usage and Social Capital

Shared contexts exist and develop among team members whenever their members have access to the same information and hence share similar content (Hinds and Mortensen, 2005). Work processes and work culture develop in a shared context. The higher the shared context, the higher will be the team’s bonding social capital. Traditional technologies are limited in conveying contextual and member-specific information and are not beneficial to the growth of shared contexts. Social media, however, can enable self-disclosure, encourage attachment, spread understanding, and communicate contextual knowledge by providing social cues through instant interaction, network meetings, and information sharing (Hinds and Bailey, 2003). These cognitive uses assist to build shared contexts and knowledge by enabling interaction and collaboration between individuals (Ali-Hassan et al., 2015). Agricultural entrepreneurs, friends, family, and entrepreneurial teams use social media tools to convey information, gather information, hold meetings, express emotions, improve mutual understanding, and develop consensus. Hence, the following research hypotheses can be postulated.

H3a: The cognitive orientation usage of social media has a significant positive effect on the bonding social capital of agricultural entrepreneurs.

The cognitive usage of SM is to produce and distribute content that improves employee profiles and features employee qualities comprising their knowledge, expertise, preferences, insights, and interests. Given that the formation of social relationships is motivated by similar activities and common interests (Zeng and Wei, 2013), the ease of generating and posting content via, combined with its collaborative nature, improves the intensity and potential for employees to build social relationships. For example, posting a manuscript, image, or video may expose an individual’s interests that will additionally increase the likelihood of establishing connections with other employees sharing similar interests. Moreover, self-disclosure of private information, photographs, stories, etc., offers chances to create new relationships between individuals. In the case of agricultural entrepreneurs, social media distribution and access to information facilitate them to identify and acquire new friends at a lower cost. Taken together, the above literature leads to the following research hypotheses.

H3b: The cognitive orientation usage of social media has a significant positive effect on the bridging social capital of agricultural entrepreneurs.

### Impact of Entrepreneurship Training on Social Media Use

Training is defined as an essential HRM procedure designed at improving individuals develop mindsets, abilities, and ideas that can enhance their job performance. It is also one of the common techniques for boosting employees’ efficiency and conveying the company’s objectives to new employees (Pham et al., 2020). Liu et al. (2019) in their research, empirically investigate the relationship between technical training and farmers’ implementation of low-carbon technology in the Hubei Province, China. Corresponding to the outcomes technical training enabled the decision-making method of implementing low-carbon technologies. Nevertheless, for technical training to be an effective channel to acceptance, farmers should be offered strong, scientific findings and pertinent knowledge of technologies, containing economic feasibility, risks, sustainability, and other possible consequences for their farm processes and finances (Gautam et al., 2017). Face-to-face training has a constant limitation related to its high expense and difficulty to logistically deliver with slight opportunity for scalability as compared to online training. Keeping in mind the impact of financial factors and time pressures on the ability of an organization to provide training to the workforce, face-to-face training is not considered a feasible alternative by many organizations, especially small and medium enterprises. This has led to the expansion and assessment of comparable training for managers and entrepreneurs delivered through an online system (Kawakami et al., 2006; Gayed et al., 2018, 2019). Strengthening farmers’ training in innovative media applications can improve their information awareness and skills in operating social media. Furthermore, farmers can learn technical expertise through training and apply this knowledge in practice, thus increasing their income and livelihood. Based on the above literature, the following research hypotheses were derived.

H4a: Significant differences exist in social media social use patterns among agricultural entrepreneurs with entrepreneurial training experiences.

H4b: Significant differences exist in social media hedonic use patterns among agricultural entrepreneurs with entrepreneurial training experiences.

H4c: Significant differences exist in social media cognitive use patterns of agricultural entrepreneurs with different entrepreneurial training experiences.

**FIGURE 1:**
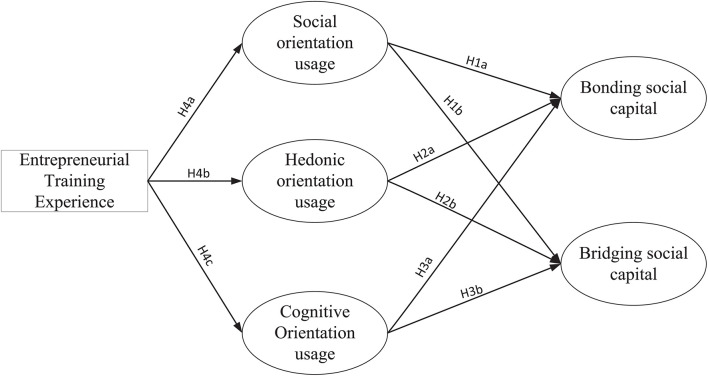
Research model.

The research model in this paper is shown in Figure 1.

## Methodology

### Questionnaire Design and Data Collection

In this study, questionnaires were distributed in the training courses of Fujian Agricultural Vocational Technology College, and, Fuzhou, Quanzhou, Jianning, and Liancheng counties and cities in Fujian during winter and summer vacations and social practice to target new vocational farmers. This research study used a convenience sampling technique for data collection. A total of 589 questionnaires were distributed and 446 valid questionnaires were collected, with a return rate of 75.72%. Among them, 50.2% were men and 49.8% were women. The number of married people with children was the largest, accounting for 69.2%, while 18.90% were unmarried and 11.06% were married without children. In terms of age, 44.3% of agricultural entrepreneurs were under 30 years old, and 43.6% were between 31 and 40 years old. In terms of education, the highest percentage of agricultural entrepreneurs were having high school (or junior college) education with a percentage of 49.8%, and the second-highest percentage were that of college graduates, accounting for 33.1%. Regarding the entrepreneurial experience, 53.12% had experience in agriculture or other fields, and 64.39% had participated in the agricultural entrepreneurship training.

Instead of requesting respondents simply whether they accept or oppose a statement, Likert scale components inquired how strongly they accept or disagree with it. The Likert scale measured the attitudes of respondents usually on a seven-point scale from 1 (=strongly disagree) to 7 (=strongly agree), with the value of 5 associated with a neutral category. Social media usage was assessed by items recommended by Ali-Hassan et al. (2015), while the items for determining were adopted from the study by Subramaniam and Youndt (2005) and Han and Hovav (2013).

## Data Analysis

### Reliability and Validity Tests

To ensure the effectiveness of the study, the reliability and validity of the scales were first tested using SPSS 24.0. The results of the reliability analysis in Table 1 show that the Cronbach’s alpha values for social use, hedonic use, cognitive use, bonded SC, and hedonic SC ranged from 0.867 to 0.914, all of which exceeded the critical value of 0.7, indicating that the questionnaire scales have acceptable reliability.

**TABLE 1:** Reliability and validity tests.

Variables	Indicators	Factor loading	AVE	CR	Cronbach’s alpha
Social use	SU1	0.808	0.597	0.881	0.880
	SU2	0.728			
	SU3	0.778			
	SU4	0.779			
	SU5	0.768			
Enjoyable use	HU1	0.798	0.646	0.879	0.879
	HU2	0.824			
	HU3	0.815			
	HU4	0.777			
Cognitive use	CU1	0.847	0.683	0.915	0.914
	CU2	0.858			
	CU3	0.803			
	CU4	0.819			
	CU5	0.804			
Combined social capital	BOSC1	0.816	0.619	0.907	0.907
	BOSC2	0.814			
	BOSC3	0.783			
	BOSC4	0.8			
	BOSC5	0.766			
	BOSC6	0.74			
Bridging social capital	BRSC1	0.796	0.567	0.867	0.867
	BRSC2	0.777			
	BRSC3	0.717			
	BRSC4	0.708			
	BRSC5	0.764			

Additionally, this study used AMOS 24.0 to conduct validation factor analysis on the variables, and Table 1 shows that the standardized factor loadings of each measurement item ranged from 0.708 to 0.858, all of which were greater than the recommended value of 0.5, indicating that each item had significant reliability. The composite reliability (CR) of the study variables ranged from 0.867 to 0.915, all of which exceeded 0.7, hence, all of them met the criteria suggested by research scholars, showing that each variable has good internal consistency. In addition, the average variance extracted (AVE) of each variable ranged from 0.567 ∼ 0.683, all of which were higher than 0.5, which met the criteria suggested by scholars, showing that each variable had good convergent validity.

Finally, this study tested the discriminant validity of each variable through the AVE method by comparing the square root of the AVE of each variable with the magnitude of the Pearson correlation coefficient between the variables. As seen in Table 2, the square root of the AVE of social use, hedonic use, cognitive use, bonded social capital, and bridging social capital were 0.773, 0.804, 0.826, 0.787, and 0.753, respectively, and the correlation coefficients between the two variables ranged from 0.412 to 0.719, all of which were smaller than the diagonal square root values, indicating that each of the study’s variables has good discriminant validity.

**TABLE 2:** Discriminant validity of the variables.

	AVE	Social use	Enjoyable use	Cognitive use	Combined social capital	Bridging social capital
Social use	0.597	**0.773**				
Enjoyable use	0.646	0.641	**0.804**			
Cognitive use	0.683	0.690	0.412	**0.826**		
Combined social capital	0.619	0.694	0.601	0.579	**0.787**	
Bridging social capital	0.567	0.646	0.482	0.717	0.679	**0.753**

*The bolded numbers in the table are the square root values of the latent variable AVE, and the values in the lower triangle are the Pearson correlation coefficients between the latent variables.*

The fit of the model was examined by AMOS 24.0, and Table 3 shows that the χ^2^/DF value is 1.417, which is less than 3, tucker-lewis index (TLI), comparative fit index (CFI), goodness of fit index (GFI), and adjusted goodness of fit index (AGFI) are all greater than 0.9, and standardized root mean square residual (SRMR) and root mean square error of approximation (RMSEA) are both less than 0.08, and the values of each fit index are within the tolerable range, indicating that the study model has a good fit and can be tested in the next step.

**TABLE 3:** Model fit.

Fitting index	Allowable range	Study model fit
χ^2^/DF	1 < χ^2^/DF < 3	1.417
TLI (NNFI)	>0.9	0.963
CFI	>0.9	0.968
GFI	>0.9	0.931
AGFI	>0.9	0.922
RMSEA	<0.08	0.036
SRMR	<0.08	0.039

### Structural Model Testing

The path relationships between the variables of the structural model were tested and the results of the path coefficients can be seen in Table 4. Social use (β = 0.432, *p* < 0.001), hedonic use (β = 0.281, *p* < 0.001), and cognitive use (β = 0.191, *p* = 0.003) had a significant impact on bonded social capital, and hence, the research hypotheses 1a, 2a, and 3a were accepted. Furthermore, social use (β = 0.195, *p* = 0.031), hedonic use (β = 0.127, *p* = 0.034), and cognitive use (β = 0.427, *p* < 0.001) significantly impacted bridging social capital, therefore, research hypotheses 1b, 2b, and 3b were also accepted. The findings of this study support the research questions of this model.

**TABLE 4:** Path analysis results.

Dependent variable	Independent variable	Path coefficients	*p*-value
Bonding social capital	Social use	0.432	0.000
	Hedonic use	0.281	0.000
	Cognitive use	0.191	0.003
Bridging social capital	Social use	0.195	0.031
	Hedonic use	0.127	0.034
	Cognitive use	0.427	0.000

### Differences in Social Media Use Patterns Among Those With Different Entrepreneurial Training Experiences

The differences in social media usage patterns among those with different entrepreneurial training experiences were analyzed using independent samples *t*-test, and from Table 5, it can be seen that in terms of social usage patterns, the mean value was 5.57 (standard deviation 1.23) for untrained agricultural entrepreneurs and 5.91 (standard deviation 0.80) for trained agricultural entrepreneurs, with a significance level of *t* = −3.20, and *p* = 0.001 < 0.05, indicating there is a significant difference in the social use pattern of agricultural entrepreneurs with different entrepreneurial training experiences, and hence supporting hypothesis 4a. In terms of hedonic use pattern, the mean value was 5.43 (standard deviation 1.22) for the untrained agricultural entrepreneurs and 5.58 (standard deviation 1.05) for trained agricultural entrepreneurs, which did not reach a significant level (*t* = −1.55, *p* = 0.125 > 0.05), indicating that there is no significant difference in the hedonic use pattern of agricultural entrepreneurs with different entrepreneurial training experiences, and therefore hypothesis 4b is not supported. In terms of cognitive use pattern, the mean value of this item was 5.37 (standard deviation 1.26) for untrained agricultural entrepreneurs and 5.69 (standard deviation 1.27) for trained agricultural entrepreneurs, and the results of the test reached a significant level (*t* = −2.76, *p* = 0.005 < 0.05), indicating that there is a significant difference in the cognitive use pattern of agricultural entrepreneurs with different entrepreneurial training experiences, and hypothesis 4c is supported.

**TABLE 5:** Differences in social media use patterns among those with different entrepreneurial training experiences.

Social media usage patterns	Entrepreneurship training	Number of cases	Average value	Standard deviation	Mean difference	Standard error heterogeneity	*t*	Significance
Social use	No	175	5.57	1.23	0.34	0.09	−3.20	0.001
	Yes	271	5.91	0.80				
Hedonic use	No	175	5.43	1.22	−0.15	0.11	−1.55	0.125
	Yes	271	5.58	1.05				
Cognitive use	No	175	5.37	1.26	−0.32	0.11	−2.76	0.005
	Yes	271	5.69	1.27				

## Discussion

The acknowledgment that digital transformation comprises features of both social and technical change is helping to encourage values of accountable innovation and other changes concerning dynamic social knowledge in research (Stitzlein et al., 2021). Nevertheless, the agricultural digital transformation can nonetheless create several issues and challenges. The usage of digital technology could have an advantage to the marginalization of the knowledge of agricultural business and but also create separation between agriculture and farmers that create issues related to the tradition of the conventional agricultural culture. Consequently, special attention should be paid to the political and social consequences of the digital transformation of agriculture. Furthermore, digitization frequently includes the usage of huge volumes of data. Therefore, safety and privacy should be completely safeguarded. In China, several farmers are unaware of this issue that online equipment necessary for digital agriculture might provide exposure to virtual hazards (Xie et al., 2021).

The results in Table 5 show that the research hypotheses of the structural model of this paper are supported and the three modes of social, hedonic, and cognitive use of SM have a significant positive effect on both bonding and bridging social capital of agricultural entrepreneurs. The explanatory power of the three usage patterns for bonded social capital was 54.6% and for bridging social capital was 56.9%, both of which were relatively strong. The use of social media by agricultural entrepreneurs has indeed led to deeper trust and affection with their team, family, and close friends, and has helped them make additional relationships beyond their current acquaintances, allowing them to expand their network and gain more weak ties, thus gaining access to more heterogeneous resources and increasing their social capital substantially.

As the findings demonstrate, farmers engage with various kinds of SC, including bridging, and bonding relations; this is somewhat related to earlier studies on SC, information exchange, and rural innovation (Parsram et al., 2012; Fisher, 2013; Tregear and Cooper, 2016; Sutherland et al., 2018). This research adds to the earlier literature by indicating that farmers create these network structures varying on their goals, capabilities, resources, knowledge, and other services. One of the most pertinent distinguishing elements appears to be individual motivations. Consequently, various kinds of farmers handle social capital types in their way. The results suggest that farmers that are inclined in the direction of executing pioneering technologies and methods are more active in exploring expert information and supplies, increasing their network from bonding to bridging SC. However, farmers that are inclined to implement established technologies and methods tend to restrict their networks. Nevertheless, irrespective of their network structure of choice, the results suggest that farmers acknowledge that participating in various kinds of SC allows them to boost corporate productivity and lessen the ambiguity linked with applying innovative technologies. This reiterates outcomes from earlier researches on farmers’ eagerness to participate in social capital (Gómez-Limón et al., 2014; Cofré-Bravo et al., 2019).

Among the three types, social interaction is the primary mode of people’s social media use. In rural social relations, the initial interaction of social media is based on the offline acquaintances’ circle of friends. Agricultural entrepreneurs leave messages to their friends, relatives, and internal teammates through social media such as WeChat and QQ, which are popular due to the convenience and low cost of social media. Furthermore, the mode of voice messages and text messages can also avoid the invariance of telephone instant communication in terms of time and expression. The frequent use of social media allows the communicating parties to get to know and be close to each other’s lives. This additionally enhances their understanding of each other’s situations while facilitating the expression of mutual care, trust, and support, making their relationships stronger and reliable. Moreover, it provides help in bringing support in terms of entrepreneurial capital, confidence, and information resources, ultimately promoting the accumulation of bonded SC. The positive promotion effect of social use on bridging SC can be explained by the Six Degrees of Separation (SDS) theory. SDS theory assumes that any two strangers as long as they pass through a particular way will definitely create a connection (Travers and Milgram, 2011). Everyone’s has interpersonal networks, and these networks will eventually form certain intersections; thus, social media offers unlimited possibilities for developing our network of contacts. In practice, agricultural entrepreneurs often meet new friends through social media communities, continuously expand weak relationship connections, and gain much useful information, thus increasing bridging social capital. In terms of hedonic use, agricultural entrepreneurs engage in recreational activities in their circle of acquaintances for leisure and relaxation, and informal communication activities within the entrepreneurial team for better relationships and meeting new people in some games and recreational activities, thus contributing to the increase of both bonding and bridging SC. The positive effect of cognitive use patterns on SC is supported by literature and practice. For example, like and comment functions, which are often used by agricultural entrepreneurs in their friends’ circle, are both interactive targeted communication (Burke et al., 2011) and increase bonding and bridging SC. Like is a kind of “relationship investment signal” (Ellison et al., 2014), which makes the recipient feel social support through simple one-click interaction and enhances the strength of the relationship between the sender and the recipient. Comments are also powerful in eliminating feelings of isolation and making the recipient feel happy and are an effective way to strengthen strong ties and promote support and encouragement. Comments can also carry personalized content, and the receiver is more likely to feel the sincerity and care from the sender, and the sender is more likely to receive further interaction and reciprocal behavior from the receiver, promoting increased trust, acquiring new followers, and bringing bridging social capital to the user compared to likes.

The results of the analysis of variance show that trained agricultural entrepreneurs have significantly higher levels of use of social and cognitive models than those who have not received training. This may be because the entrepreneurial training process includes a lot of training in e-commerce entrepreneurship and social media usage skills, which can improve the level of scientific use awareness and operational skills of entrepreneurs. Although this research signifies a preliminary glance at this crucial part of Internet usage, it does indicate possible strategy controls to improve the technical capability, consciousness, and usage of the Internet and social networking sites by agricultural entrepreneurs, mainly for improvement drives. Previous research has underlined that entrepreneurs confront numerous hurdles in the launching procedure containing lack of business knowledge (Fairlie and Robb, 2009), smaller amounts of financial investment, and a need to stabilize family obligations. Intern applications and social networking site usage can also be a hindrance that entrepreneurs must conquer (Mack et al., 2017). Possibly the most crucial element in describing the diversity of social media usage was previous entrepreneurial knowledge. This conclusion decreases from variations in intellectual frameworks between beginner and skilled entrepreneurs that influence their capability to identify prospects and react to technological transformation (Baron and Ensley, 2006). Skilled entrepreneurs with stronger cognitive frameworks are in a superior situation to develop innovative Internet applications and social media using techniques as compared to beginner entrepreneurs possessing narrow cognitive frameworks.

## Theoretical and Managerial Implications

This study examined the relationship between different modes of SM use and SC and concluded that (1) all three modes of social media usage including social media use, hedonic use, and cognitive use significantly contributed to the positive accumulation of social capital, and agricultural entrepreneurs can increase their social capital by using social media; (2) entrepreneurship training can significantly improve the level of social media use of agricultural entrepreneurs. The findings of this research represent a new empirical contribution linking SM, SC, and entrepreneurial training. This research study provides a theoretical model to measure the impact of three dimensions of social media usage including social, hedonic, and cognitive orientation usage with two dimensions of consisting of bonding and bridging social capital adapted from social capital theory. Furthermore, this study also explores the relationship between entrepreneurial training and social media usage.

The findings of this paper have implications for the use of social media in agriculture. First, agricultural entrepreneurs should fully recognize the role of in entrepreneurial activities and take the initiative to fully utilize the social and cognitive functions of social media to acquire and accumulate social capital and provide support for entrepreneurial activities. Second, the government can carry out agriculture-related entrepreneurship education in rural areas to train entrepreneurs’ social media operation skills level, create conditions to improve farmers’ media literacy, and provide full opportunity to the powerful role of modern information technology for rural revitalization. Furthermore, according to the findings of this study, it can also be inferred that training can enhance agricultural entrepreneurs’ confidence level which will, in turn, increase their communication capabilities in utilizing electronic platforms. Agricultural entrepreneurs can foster innovations in their farming styles, farming configurations, and farming experience by creating and implementing social media networking strategies.

## Limitations and Future Research

In terms of limitations to this study, the data were collected from a specific country, results could differ in other circumstances, particularly in the institutional setting. Moreover, this study is focused on the viewpoint of respondents. Consequently, this viewpoint could exceed or underestimate the actual part of social media usage on social capital. Potential researchers can utilize the study model in a diverse country setting following a distinct culture. Because this study utilized the model in an emerging country, potential researchers can utilize the study model in a developed economy and evaluate the outcomes centered on the comparison in terms of usage of social media usage in an emerging and developed economy.

This study introduces SM research into the field of agriculture-related entrepreneurship, focusing on the impact of different SM usage patterns of agricultural entrepreneurs on social capital, which is an addition in the literature of SM research, but the relationship between SM usage and SC may also be influenced by personal and environmental factors of entrepreneurs. Therefore, future research can further explore the moderating factors, such as the gender of entrepreneurs and the nature of media, to enrich the content of social media research in agriculture.

## Data Availability Statement

The raw data supporting the conclusions of this article will be made available by the authors, without undue reservation.

## Author Contributions

G-HX and L-PW: conceptualization, methodology, and formal analysis. AK: software and visualization. L-PW: validation and supervision. G-HX: investigation. G-HX, L-PW, and AK: writing—original draft preparation and writing—review and editing. All authors have read and agreed to the published version of the manuscript.

## Conflict of Interest

The authors declare that the research was conducted in the absence of any commercial or financial relationships that could be construed as a potential conflict of interest.

## Publisher’s Note

All claims expressed in this article are solely those of the authors and do not necessarily represent those of their affiliated organizations, or those of the publisher, the editors and the reviewers. Any product that may be evaluated in this article, or claim that may be made by its manufacturer, is not guaranteed or endorsed by the publisher.
